# Unravelling Barriers to Accessing HIV Prevention Services Experienced by African and Caribbean Communities in Canada: Lessons from Toronto

**DOI:** 10.5539/gjhs.v4n3p1

**Published:** 2012-05-01

**Authors:** Paulson Amibor, Ayodeji Bayo Ogunrotifa

**Affiliations:** 1 International Public Health Consultant, Geneva, Switzerland; 2 School of Social and Political Sciences, University of Edinburgh, UK

**Keywords:** HIV-prevention services, Afro-caribbean, community-based organisations, people living with HIV/AIDS (PHAs), thematic analysis

## Abstract

Barriers to accessing HIV-prevention services, experienced by African and Caribbean communities in Canada, is an issue warranting sustained research. This study seeks to achieve a better understanding of the nature of HIV-prevention services in Canada, and to explore the dynamics, which underpin barriers to accessing these services confronting African and Caribbean populations in Toronto (Canada). This study also endeavours to assess what is being done to reduce these barriers. Semi-structured qualitative interviews with 7 professionals and community workers who were involved in organizing, researching and delivering HIV-prevention services were conducted for this study. Four themes pertaining to barriers to accessing HIV-prevention services, including, levels of cultural competence and sensitivity among service providers; cultural and social stigma directed at persons living with HIV/AIDS; various social determinants of health, including gender, race and precarious immigration status’; as well as constrained funding resources that are available for service providers; were uncovered in the findings of the study. The paper concludes that several health promotion and health education initiatives exist, which can help reduce these barriers to HIV-prevention service access for these populations. However, in order to ensure their effectiveness there will be much needed involvement from community and other relevant government agencies, which will need to work separately and in conjunction with one another, in order to tackle some of the broader issues that affect these populations.

## 1. Introduction

### 1.1 Introduce the Problem

HIV prevention is crucial for the long-term control and management of the global HIV/AIDS epidemic (Ng et al, 2011). Results of small-scale studies have shown the potential of different strategies in HIV prevention (Ng et al, 2011). Across the world, a small but growing number of countries have reduced HIV prevalence through sound prevention efforts (UNAIDS, 2005). This has had a counter effect on the reality that high rates of HIV transmission have resulted largely from the failure of populations to use available and effective prevention strategies and tools, as well as due to poor coverage levels of HIV prevention programmes (UNAIDS, 2005). The latter has been reaffirmed by the fact that HIV-prevention services still don’t reach most of those in need (HIV Prevention Working Group, 2009). Against this backdrop, and despite the fact that sound prevention and treatment efforts often translate into gains being made in reduced levels of new infections over time, the HIV epidemic in North America and Western Europe have remained stubbornly steady, despite universal access to treatment, care, support and widespread awareness of the epidemic and the causes of HIV infection (UNAIDS, 2011). Thus, HIV incidence within these countries has changed little since 2004 (UNAIDS, 2011).

Additionally, within some of these countries, new infections among populations and particularly among specific sub-groups, such as some ethnic minority groups, have not only remained steady, but have in some cases exceeded those of the general population. For example, when compared to general populations living in Western developed countries, such as Canada, the United Kingdom, France and Germany; black African and Caribbean communities have continued to experience higher rates of HIV infection (ACCHO, 2006). Despite these circumstances, measures that might be taken by these communities to counter these trends and deal with HIV/AIDS, and the related stigma and discrimination often concomitant with the disease, have been complicated by other factors such as, gender, discrimination due to race, immigration and refugee status (ACCHO, 2006). Also, in the case of Canada in particular, where many efforts have been made to mitigate the impacts of these challenges, some of these issues have nevertheless continued to persist. This has remained a dilemma that has also been particularly pronounced in the Greater Toronto Area.

Moreover, the Canadian approach to HIV/AIDS, which recognizes many of these challenges, has been characterized as being grounded in human rights and social justice, aiming to protect the most vulnerable of populations. In light of this, evidence and population-based approaches have been central to assessing the needs and priorities of the national AIDS response in terms of research, policy and program development, and monitoring and evaluation measures (UNGASS, 2008). Part and parcel with this Canadian response has been the role that community-based initiatives (especially involving civil society) have played in this regard, which have been instrumental in mounting a vigorous response to the challenge of HIV/AIDS. With resource support from government and across society, community organizations continue to play a key role in designing and delivering front-line services, identifying emerging policy issues, and developing appropriate policy responses. Community organizations participate in national planning and expert panels, the development and championing of innovative approaches in prevention and support, and in the delivery of programs (UNGASS, 2008).

In Toronto, community-based strategies have helped in responding critically to HIV/AIDS (Toronto Public Health, 2006). Some of these achievements have been particularly evident within the context of the city’s efforts to tackle local inequities in light of the larger global HIV/AIDS pandemic. Despite such successes, however, a closer look at the HIV situation in Toronto reveals that those same services and resources are not easily accessed by some of the people who need them most. These conditions have been particularly pronounced among women in the Afro-Caribbean communities who now account for the largest amount of new infections among this sub-population within the city (Black CAP, 2009). It is surmised that one of the factors that has precipitated this disturbing situation, has been due to the underutilization of HIV-prevention services by this population. It was the consideration of some of these realities that gave us the impetus to undertake this research, and to assess efforts being made by community-based and affiliated organizations in trying to address these challenges.

It is our contention here, that significant improvements in the uptake of relevant and available HIV-prevention services will play a key role in averting new HIV infections, as well as in curtailing the growing preponderance of transmission levels among the African and Caribbean constituencies in the Greater Toronto Area. Despite the existence of studies that analyze the implications of the use of HIV-prevention services in Toronto, assessments made of the barriers to accessing these services by African and Caribbean communities in Toronto has been largely under-studied - especially considering the magnitude of transmission patterns within these communities. This study seeks to fill this gap by uncovering the barriers associated with accessing these HIV-prevention services, with a view to offering practical policy and programmatic measures that will assist in ameliorating this situation.

### 1.2 Patterns and Trends of HIV Incidence and Prevalence Levels among African and Caribbean Populations in the Greater Toronto Area

Statistics collated by the Black Coalition for AIDS Prevention (Black Cap) (2009), noted that since the year 2000, rates of HIV infection in Toronto’s black community have more than doubled. Black—African and Caribbean persons, account for more than one-fifth of all new infections in Toronto, compared to the early nineties when this population only comprised one-tenth of new HIV infections. Additionally, two-thirds of these infected individuals have been identified as black males (Black Cap, 2009). Similarly, black women are increasingly at risk of HIV infection and now represent about two-thirds of women infected with AIDS in Toronto (Black CAP, 2009). Evidence examining the prevalence levels of HIV among this community, indicate that although accounting for only 8.4% of the city’s population (City of Toronto, 2006), 15.9% (99/625) of HIV cases diagnosed in Toronto in 2007 were among its black population (Falconer, 2008). These realities can be seen as compounded by findings of studies that suggest that about half of the African, Caribbean and Black populations in Ontario who are living with the HIV virus are unaware that they have it (Black Cap, 2009).

## 2. Method

This study employed qualitative methods. Qualitative research was selected as the mode of inquiry because of the aim of obtaining first-hand feedback on the experiences, perceptions, attitudes and beliefs of the interviewees. These included numerous professionals and community workers from community-based and other organisations who worked directly as HIV-prevention service providers, researchers, organizers, and as advocates for HIV prevention, treatment, care and support service-use. These services were available for use by all residents of the Greater Toronto Area. According to the 2011 Canadian National Census, at the time this study was conducted, the Greater Toronto Area had a population of 5,555,912 persons (Statistics Canada, 2012). As such, all of these persons would have been eligible to seek services from these providers. Albeit, other HIV-prevention services for residents of the Greater Toronto Area did exist within this jurisdiction at this time, and therefore residents of this region were not limited to the uptake of the services being provided by the service providers who were the subjects of this study.

Furthermore, this work was done with the aim of collating insights into what the service providers deemed as barriers to HIV-prevention service uptake by the population of interest; gaining a greater understanding of what the service providers perceived as successes and failures of the work they did; and to help identify gaps that could be filled by the present study, as well as by future research. This qualitative research project is based on semi-structured interviews with these professionals and community workers. Convenience sampling was used to select these participants, which include 7 individuals from the Greater Toronto Area of Canada.

We chose to centre our study primarily on community-based, non-profit organizations responsible for the delivery of HIV-prevention services in the Greater Toronto Area. These services included external and in-house prevention related programmes and activities. Some of these included community outreach programmes and counselling services, as well as health promotion and media campaigns and initiatives, for adults and youths. Utilizing a variety of delivery mechanisms, some of these services were carried out directly within communities, while others were performed at both major and more local municipal events.

Similarly, we chose a community-based approach to our study, because of the long standing tradition in Toronto, which acknowledges that a community-based strategy is critical for responding to HIV/AIDS. The city of Toronto also has a history of strong community leadership in the response to HIV/AIDS.

### 2.1 Interviews

Using the interview technique, a semi-structured interview format was chosen for this study in order to confer it with the benefits afforded by both structured and unstructured interview methods. Semi-structured interviews provide greater breadth or richness in data compared with structured interviews, and allow participants freedom to respond to questions and probes, and to narrate their experiences of HIV-prevention services without being tied down to specific answers (Morse & Field, 1995). A further advantage they have over unstructured interviews is that they allow for comparisons to be made across interviews, given that some of the questions they contain are standard (Minichiello et al., 1999).

### 2.2 Data Analysis Techniques

This study uses thematic analysis as the basis for the data analysis strategy. Thematic analyses assist in the search for themes that emerge as relevant to the phenomenon under description. The process provides the opportunity for identifying themes through careful reading and rereading of the data (Ezzy and Rice, 1999: 258). It is a form of pattern recognition within the data, where emerging themes become the categories for analysis (Fereday and Muir-Cochraine, 2006). The data analysis process was directed towards discovering patterns and the shades of qualitative meaning that emerged from the transcriptions. The analysis was characterized by an intensive dialogue with the text. An understanding of the phenomenon under investigation was sought where the whole was understood in terms of its details, and the details in terms of the whole.

In this research, a systematic method of thematic data analysis was adopted, as informed by the work of Titchen and colleagues (Edwards & Titchen, 2003; Titchen, 2000; Titchen & McIntyre, 1993). This method allowed for the systematic identification of participants’ interpretations and constructs (first order constructs), which were then layered with the researchers’ own understandings, interpretations, and constructs (second order).

This study specifically aligned with Aronso’s (1994) pragmatic view of thematic analysis, and thus helped to develop four stages in the analysis (see Table below). Throughout all stages of the data analysis, there was ongoing interpretation of the research text and the dynamics of the HIV-prevention services targeting the African and Caribbean communities in Toronto. This was done in order to observe how successful service providers have been in reaching this population. By constantly cross-checking our interpretations with the original transcripts, we sought to maintain closeness (or faithfulness) to the participants’ constructs, grounding our interpretations in the data. This strategy for maintaining authenticity was suggested by Lincoln and Guba (2000). Dialogue between the authors of this paper about emerging findings served to further check the faithfulness or authenticity of the data. However, the identities of the participants were concealed in order to maintain the confidentiality of the information obtained.

**Table 1:** Stages of the analysis

STAGES	TASKS COMPLETED
Stage one: Transcribing and Organising the interview text	This involved the transcription of interview data already collected. This stage helps to organise already collected data into texts, which gave much impetus to the preliminary reading and interpretation of texts to facilitate coding.
Stage two: Organising patterns of experiences	At this stage, through preliminary reading and interpretation of the texts to facilitate coding, patterns of experiences can be listed such that all data that relate to the already classified patterns are identified.
Stage three: Combining all related patterns into sub-themes	This stage helps to catalogue all related identifiable patterns into sub-themes. Sub-themes that emerge from the informants’ stories are pieced together to form a comprehensive picture of their collective experiences.
Stage Four: Developing Themes	This stage involves grouping sub-themes into themes. In developing themes, further elaborations of themes were made by building valid arguments for choosing the themes. This is done by reading the related literature.

#### 2.2.1 Stage One: Transcribing and Organising the Interview texts

The interviews, which were audio-taped, were transcribed into textual material. The texts were then constructed for each participant. The authors read and re-read all written texts for each participant in order to become very familiar with the text sets. Dialogue between the researchers served as a vehicle for reflecting on emerging ideas, and was also a means for developing and expanding these ideas. Such dialogue is valuable for providing insights, considering alternative interpretations and contradictions, and for thoroughness in interrogating the data.

#### 2.2.2 Stage Two: Organising Patterns of Experiences

The next step involved the identification of patterns of experiences that formed the basis for the thematic coding of the data extracted from the interviews. Through the preliminary reading and interpretation of texts to facilitate the coding process, the patterns of experiences of the participants were identified. The transcriptions were read through several times to ensure that all patterns of experiences deemed most meaningful and relevant to the research were picked up and assessed. This process was done extremely meticulously in order to ensure that the statements selected to substantiate the emerging sub-themes were as germane as possible, as well as to ascertain that they would be coherent with the other excerpts chosen.

#### 2.2.3 Stage Three: Combining all related Patterns into Sub-Themes

All of the already classified patterns were coded into sub-themes. Sub-themes here represent what Titchen & McIntyre (1993) called, First Order Constructs where ‘participants ideas were expressed in their own words or phrases, thus capturing the precise detail of what the person is saying’. These constructs were related to the research questions linked to the dynamics around HIV-prevention services among African and Caribbean populations, which are addressed in this article. First Order Constructs were identified first for all participants in the study, with a constant process of checking for the appropriateness and completeness of these constructs. The texts were coded into a computer to identify these constructs.

#### 2.2.4 Stage Four: Developing Themes

Various sub-themes that emerged from already classified patterns were developed into themes. Themes were developed from the results of stages one to three of the analysis.

In developing the themes, further elaboration of the themes were made by building a valid argument for choosing the themes. This process is what Alfred Schultz (1967) described as Second Order Construct. The Second Order Construct files were grouped together into a smaller number of broad themes according to the interviews. In this stage, themes and sub-themes were further elaborated and their relationship clarified by reading and re-reading all the data. For instance, sub-categories or sub-themes such as gender inequalities, racial and ethnic discrimination and immigration status were observed as significant considerations that should be developed into one of the four major themes (elaborated further in the Results section below). These themes corresponded with perceived barriers to accessing HIV services being experienced by the African and Caribbean populations.

### 2.3 Demonstrating Quality and Reflexivity in Qualitative Research

Seers and Toye (2012) have asked ‘what do you need to look for in qualitative research to show that the study is of high quality?’ Several authors like Silverman (2001) and Seale (1999) had earlier asserted that the quality criteria in qualitative research should be demonstrated in terms of validity and reliability, while others like Koch (1996), Koch and Harrington (1998), and Leininger (1994) have argued that evaluating quality in qualitative research should be based on the philosophical and methodological assumptions on which the research is based. However, we surmise that evaluating quality in terms of Public Health and Health research should be based on the validity and reliability of the research. Hence, we aligned with Silverman (2001) and Seale (1999), because we felt that qualitative research should be able to be replicated, if the credibility of the methods are to be guaranteed. Validity in this regard means truth (Silverman, 2001) or the extent to which the reader judges the procedure for arriving at the conclusion to be truthful. In the quest of demonstrating validity, the rigorous use of systematic methods of data collection and analysis, as well as transparency in documenting these methods has to be made clear for the reader to scrutinise.

This is the rigour with which this study has undergone. We achieved this through a 3 month data collection process from the study area, which was collected between the period of May and July 2009. Since then, we have made many painstaking efforts to analyze the data so as to reflect the truthfulness of what the data conveys. Interviews were conducted with key professionals and researchers in community-based and affiliated organisations who have knowledge and experience with health and other social issues affecting the African and Caribbean populations in the Greater Toronto Area. This also allowed us to follow a semi-structured interview method, and use open-ended questions to facilitate informal discussions.

In terms of reliability, we have been able to demonstrate the process of our data analysis systematically, which other scholars can repudiate. This is demonstrated in the methods section above.

During the data collection and analysis process, we strived to be as reflexive as possible. This was done first, so as not to bring biases to the interviews while conducting them; and second, in order to avoid undermining the analytical process. As researchers, we dialogued constantly about the initial patterns the data were taking, as well as on the emerging sub-themes and themes, in order to ensure that what we were coding was a reflection of what transpired in the field. Such dialogue helped to provide insights, reconcile any possible disagreements, and enable us engage critically with the data. Therefore, critical debate of the four themes, coupled with a thorough literature review of data corresponding to these themes, bolstered our understanding of the phenomenon under consideration.

## 3. Results

Several themes emerged that elucidate the perceived barriers to accessing HIV-prevention services experienced by Toronto’s African and Caribbean populations. The themes are presented here, and are corroborated by responses received from the participants in order to demonstrate grounding in the data.

### 3.1 Theme 1: Cultural Competence and Sensitivity among Service Providers

A key finding common to all the participants was that a lack of cultural competence and poor identification with the communities being served on the part of HIV-prevention service providers, could often act as a barrier to the uptake of these services by the target populations. This was described as a factor that often characterized many generic HIV-prevention services that were available to populations in the Greater Toronto Area.

Assuming that the only difference would be that we try to cater to the different languages, because a lot of the African populations…they speak their own mother tongues and we have different staff that work here that speak those languages, so in terms of language barriers there are no real language barriers here because we speak most of the languages that would be the only difference between us and another government organization they would not have the cultural sensitivity to go by (Interviewee 3, Paragraph 14)

*African communities all have their own traditional, cultural and religious backgrounds in sex, sexuality, and regarding HIV/AIDS. So it is not easy to penetrate and educate African communities like other communities on issues around HIV/AIDS, sexuality and other aspects related to HIV. But, as I have told you, our strategy addresses all of those issues that exist within the African community, so we tailor for specific groups (Interview 6, Paragraph 10)*.

Of course first of all I will tell you what, because we will be culturally appropriate and competent, because we are talking about ourselves, and so I will know the issues that impact my life because I am part of that community…but we also have to remember that we are not homogenous, we all have, cultural, religious and linguistic differences, so we will still have different approaches, which are culturally appropriate and sensitive (Interviewee 4, Paragraph 10)

One of the key findings here is that the necessary measures that will be required to meet the African and Caribbean communities with appropriate HIV-prevention services may need to vary given some of the cultural needs of these communities vis-à-vis those of other sub-populations or community groups. These differences were found to be extant not only between the African and Caribbean communities and other communities, but also within African and Caribbean communities themselves. The interviewees were cognizant of this, and realized that conceiving of these communities as homogenous, could pose difficulties with both optimizing the design of relevant services, as well as the subsequent delivery and uptake of these services. The service providers continued to indicate their efforts to be conscientious in this regard.

### 3.2 Theme 2: Stigma

A key determinant that was perceived to also play a role in deterring these communities from the uptake of HIV-prevention services, were the fears of being identified by other members of the community while doing so. The interviewees indicated that in their experiences, they felt as though many members of the community had adhered negative connotations to HIV/AIDS, and this was thought to result in a lot of stigma being exercised towards individuals who were thought to be living with the condition.

Stigma is one of the biggest things, because I will tell you what once one identifies that this place is an AIDS Service Organization (ASO) most of them shy away, and also, they don’t necessarily go to ASO’s in their neighbourhoods…, and if they know someone from their community goes to the same clinic or centre they won’t go (Interviewee 4, paragraph 4)

Certainly with our community there is the big issue with privacy and confidentiality and stigma and this is an experience and I think that there is this myth that black people will always walk into an organization and not feel that weird, but the reality is that our organization deals with a lot of that and the reality is that a lot of our clients will not come here, they won’t, why because this space in our community has a lot of connotations, so if they walk in here people are going to assume that they are HIV positive or whatever (Interviewee 1, paragraph 8)

And other issues like stigma and discrimination also, so people will not go for testing, because if someone is tested and they know and it turns out to be positive, and even without being positive by going to visit a centre or a voluntary treatment and counselling centre will mean something to the community. So you’re part of the community, so you have to meet with the norms and the traditions of the communities, so this complicates the decisions around testing and around disclosure (Interviewee 6, paragraph 13)

…for reaching the community, so the main difficulty is in building trust with the community, because there has been a lot of stigma, not there has been, there still is a lot of stigma surrounding people who are infected or affected, who is at risk for it, that alone may kind of even shut people down from wanting to even engage or talk about the topic or even talk about their risk factors for it (Interviewee 5, paragraph 14)

These findings clearly represent an area in which more concerted efforts can be made at both the community and government levels within the wider remit of public health, in order to address some of these impediments.

### 3.3 Theme 3: The Realities of the Social Determinants of Health

Throughout the study, key underlying factors that were continuously referred to when identifying perceived barriers to service access among Afro-Caribbean communities, included: the impact that various social factors or *social determinants of health*, had in this regard. Several determinants were described, which were reiterated in some form or another by the participants.

So the general issues are the social determinants of health, immigration, poverty, lack of integration, culture, religion and taboos surrounding sex, sexuality, HIV/AIDS and also gender issues. There is huge gender inequality and economic power inequalities within the African communities (Interviewee 6, Paragraph 20)

Race and racism and these are key issues, which affect people’s abilities to access health care and people’s abilities to access other institutions and other carers that would help reduce their risk for HIV. We recognize racism as a determinant of health, and that is very foundational to our work (Interviewee 1, Paragraph 19)

I think that newcomers as a whole are a vulnerable group, obviously as you know even black people who are born here have their own problems about accessing health services and challenges about accessing health info etc, and newcomers experience that doubly…immigration status, lack of status, plays a role in that really being a newcomer period; and their risk largely depends on themselves largely not being able to assert themselves, and a sense of agency around their health, and so the locus of control is in others, so either that is in my immigration officer or it is in my lawyer (Interviewee 7, Paragraph 12)

*…obviously a lot of members from our community face issues around race and racism, but also a lot of lack of understanding or expertise of certain issues about immigration or whether its about other issues in relation to immigration, like race and employment* *(Interviewee 1, Paragraph 14)*

… factors surrounding sex and sexuality, and also gender issues, gender discrimination, gender inequality, and gender economic empowerment within the African community…I think the main ones are like I said, cultural issues are one thing, and people have kept their own cultures of their origins regarding sex negotiations, safer sex practice, discussions around sex and sexuality and related areas, which hugely affect the ability of partners to negotiate on prevention methods, on condom issues and other issues (Interviewee 6, Paragraph 18)

…straight (African and Caribbean) women are increasingly likely to access services related to HIV and AIDS… then there is the issue of culture and gender, I think that women are much more likely to access support and this gets back to masculinity and femininity and I know these things are socially constructed, but women are much more likely to use services and health care than their male partners (Interviewee 1, Paragraph 11)

The findings of this study revealed that social determinants of health such as racism and discrimination, immigration status, gender-related issues—such as gender inequality and discrimination, and gender differences in health seeking behaviour in the Afro-Caribbean communities, are perceived to act as barriers impeding HIV-prevention service uptake.

### 3.4 Theme 4: Insufficient Resource Allocations

A perceived significant factor that was felt to serve as a barrier to reaching the Afro-Caribbean populations with HIV-prevention services was the issue of a lack of resources. It was observed that this lack of resources, which typically translated into the under-funding of some of these existing services, often also led to the under-staffing of, and the poor geographical distribution of available services for those in need. It was felt that this resulted in the subsequent underutilization of these services, which were available for meeting the needs of African and Caribbean populations.

…so I love this organization…so the work environment is good, the drawbacks are it is very stressful, so we talked about going beyond, going above and beyond the client…after a while it can get kind of tiring and get kind of stressful, trying to gauge, I mean we’re a non-profit organization so our budget is not that big, so in terms of being able to make and do different things with less, that type of situation, which can be a strain because it often means that you take on several different roles simultaneously to get things done on time or in a timely fashion (Interview 5, Paragraph 17)

…in terms of services being available in those communities, a lot of the communities in the west-end and in Scarborough and in Malvern are underserved; there is a lack of HIV health services in Toronto, I mean there is a huge concentration in the centre downtown core and in the west-end and east there’s one in almost every almost like a hundred miles it seems, in relationship right, so the lack of services…even the transportation services in those areas are very poor like you’ve been to Scarborough (chuckling) once every half an hour the bus comes, its kind of hard to deal with (Interview 5, Paragraph 15)

For example within the support department we have or we had until very recently one support coordinator and she was basically doing the job of five people (Interview 2, Paragraph 13)

In terms of planning…if something comes up, because you know there is this perception that there is this somewhat of an under-representation you know some communities are sometimes over-represented and some communities are underrepresented, so this brings some kind of funding issues to the fore, but we are still working on some resource mobilization (Interview 6, Paragraph 23)

These findings suggest that not insignificant barriers impede service providers from reaching these populations, and that the existence of scare resources may play an important role in this as well.

## 4. Discussion

This research has uncovered the critical need for a better understanding of the nature of HIV-prevention services, and for the need to explore the dynamics, which underpin barriers to accessing these services that are experienced by African and Caribbean communities residing in the Greater Toronto Area of Canada. The results of this study are drawn from field work conducted in the Greater Toronto Area. The findings of the study show that the level of cultural competence and sensitivities among service providers, social stigma, the complexities rendered by myriad social determinants of health, and the challenges of under-funding and inadequate resources, contribute to barriers impeding these populations from the uptake of available HIV-prevention services. From the findings, it can be observed that many generic and some targeted services exist in Canada that are readily available for use by African and Caribbean communities, but that the above factors, sometimes operating independently and other times working in conjunction with one another, often deter these populations from accessing these services.

This study revealed that one of the major factors impeding service uptake was the degree of cultural incompetence exhibited by the service providers. This finding is corroborated by Ellen Wahoush (2009) who asserted that cultural competence and sensitivity within health care and other settings should be the medium through which professionals meet the needs of the diverse populations that they serve; through the use of communication skills both verbal and non-verbal, and training whereby professionals gain knowledge about the values and beliefs of the people they are serving. Similarly, researchers at CDC (2007), identified that in order for HIV education interventions to be successful, they (service providers) must be culturally competent. Noting that cultural competence begins with professionals understanding how the clients’ cultures affect their beliefs, perceptions, attitudes, behaviours and how they relate to exposure to HIV and STDs.

With respect to the latter point, this may be particularly significant given that one’s attitudes and behaviours may not only affect one’s approaches towards service providers and service uptake, but may also affect an individual’s perception of their levels of risk of exposure to HIV. Such observations may also have implications for the Afro-Caribbean populations in the Greater Toronto Area, as well.

Findings from a *United Nation’s (UN)* *Interagency task team on HIV* (2008), also substantiated some of our findings from the field, which suggested that community-based approaches to promoting HIV-prevention services build on shared values and norms, belief systems and social practices, which permit culturally sensitive discussions of HIV and sexual and reproductive health. A thorough understanding of common values and belief systems also helps to identify positive values and practices that can facilitate and more effectively promote HIV interventions (IATT, 2008). Thus, cultural knowledge, awareness and engagement of local communities are vital in advancing effective and sustainable change (IATT, 2008). These notions were continually reiterated by the service providers interviewed. This was especially so within the context of the emphasis they placed on the need for cultural competence and sensitivity on the part of the HIV-prevention service providers, which they felt should be employed within their efforts to meet the needs of the Afro-Caribbean populations.

A UNAIDS policy position paper also supported some of our findings. It indicated that, what was of high importance in a comprehensive approach to HIV prevention was that it must address not only risk, but also deep-seated causes of vulnerability, which reduce the ability of individuals and communities to protect themselves and others against infection. This necessitates for instance greater opportunities and greater equity in education and employment for women, young people and marginalized populations, who are particularly vulnerable to HIV (UNAIDS, 2005).

The need for additional resources being made available for service providers was also an area of concern that was raised by the interviewees in this study. Insufficient resources were identified as a shortcoming of some of these services, and were seen as a factor stifling the extent to which the reach of available services could be optimized.

Also, many of the observations that were described within this paper and which were identified as key barriers to the uptake of HIV prevention services, were barriers that were found to be prevalent within the African and Caribbean communities themselves, as opposed to with the service providers. This should also do much to inform the approaches, and indeed places emphasis on the enhanced role and capacity that community-based organizations will have to assume in both the targeting and meeting of the needs of Afro-Caribbean populations.

## 5. Limitations

In a discipline with many quantitative orientations, such as Public Health, the small sample size (of participants) used for this study might be seen as a limitation. This is particularly in regards to the potential limitations that might be imposed on the ability to generalize the study’s findings to other ethnic minority populations or sub-groups within the Greater Toronto Area - or even perhaps other settings in Canada. While qualitative researchers downplay the use of large samples, their emphasis on using small pieces of samples hinge on their perceived sufficiency for the analysis and their application to social realities. Silverman (2001) has illustrated how this distinction is particularly salient in qualitative research. He argued that the underpinnings of qualitative research are, ‘the quality of the analysis rather than how large the recruitment of the sample’ (Silverman, 2001). The use of small samples - often a feature of qualitative research - in this study helped to uncover and elaborate some perceptions and viewpoints, which would not have been able to be as thoroughly delineated had we employed quantitative approaches to our analysis. Therefore, using qualitative methods makes some of this study’s findings richer, more iterative and more significant.

A further limitation in this study was the fact that the youth populations among the Afro-Caribbean constituencies in Toronto are not thoroughly addressed in light of barriers they experience when trying to access HIV-prevention services. The relevant experiences of these youth were given a lot of mention in both the field work, as well as in the supporting literature that was reviewed. Perhaps, a specific focus on this sub-group can be addressed exclusively in subsequent studies.

Another obvious limitation of this paper is that the interviews were conducted with HIV-prevention service providers, vis-à-vis people living with HIV/AIDS (PHAs). This can be seen as a drawback given that first-hand feedback obtained from individuals living with HIV/AIDS, could have provided more intimate and holistic insights into some of the barriers to service access that they experience. However, although such a scenario might have been ideal, it might have still engendered its own limitations. For instance, had interviews been conducted with PHAs, this would not negate the fact that the potential for respondent bias is inevitable in all qualitative interview research, and that such bias might have therefore been equally introduced into a study in which PHAs were interviewed. That said, this may similarly have also been the case in the present study. However, through the use of our systematic methodology and reflexive approaches to the interviews, it is hoped that such biases were kept as minimal as possible.

## 6. Conclusions

The increasingly high new rates of HIV infection that continue to be experienced by African and Caribbean populations in the Greater Toronto Area, and especially among Afro-Caribbean women in this jurisdiction, remains concerning. As the Afro-Caribbean community are unequivocally part of the larger Greater Toronto Area’s population, this issue qualifies as a significant public health concern.

It also remains concerning that the uptake of HIV-prevention services, which have been deemed an effective and practical way to significantly reduce HIV transmission levels, have been observed to be largely underutilized by these communities. The reasons for these poor user habits are perceived to stem from: poor cultural competence and sensitivity among service providers; stigma associated with HIV/AIDS that were perceived to be somewhat prevalent within the Afro-Caribbean community; social determinants of health, manifested through factors such as social under-empowerment due to poor immigration status, gender, and race; and issues such as the insufficiency of resources that were available for service providers to optimally carry out there services. These factors constitute impediments (or barriers) that may affect the access these communities have to the uptake of HIV-prevention services.

Furthermore, both the field work and the secondary research conducted for this study point to the value of the work being done by community-based organizations, and see these operations as requisite for yielding an effective AIDS response among the Afro-Caribbean community. Efforts made in this regard, however, should certainly be evidence-based, community-driven and should reflect the interests of stakeholders and all other entities involved.

Efforts should also be made to make existing generic HIV-prevention services more culturally sensitive and competent, and those organizations that exclusively deal with the Afro-Caribbean community should have their services strengthened and bolstered so that their efforts can be taken to scale. In addition, Afro-Caribbean communities, as well as local municipal, and other relevant government bodies and actors, should work in synchrony and forge solidarity to see to it that such barriers can be surmounted.

## 7. Recommendations

Based on the findings of this study, several potential recommendations can be made for reducing barriers to accessing HIV-prevention services experienced by Afro-Caribbean communities in Canada. Some of these recommendations may also have applications for prevention measures or initiatives that might also be employed for other minority populations, also significantly affected by HIV/AIDS. First, enhanced training of staff regarding issues of cultural sensitivity and competence can be very effective. Such training could also be expedited by the provision of more culturally diverse services, as well as the hiring of staff which represent a wider array of individuals from different ethnic and cultural backgrounds – including individuals from backgrounds similar to those of the communities in which the greatest need for prevention services exist.

Second, necessary measures to reduce HIV/AIDS-related stigma within the Afro-Caribbean community will have to be pursued with some degree of vigour. This can most appropriately be done at the level of the community through health promotion and education initiatives that create spaces for discussions and which clarify issues around the dynamics of HIV risk and prevention.

Third, it should be kept in mind that devising measures for addressing challenges engendered by social determinants of health can often be less straightforward. This can often be due to the fact that such issues are often mired and interwoven within hierarchical networks of power, which are often manifested through complex arrays of institutional and systemic disparities in distributions of power. In a similar respect, this study’s findings indicate that measures to tackle these factors will have to be inter-sectoral and interdisciplinary in nature, and will have to ensure that actors in many settings are aware of the implications that their work and services (as well as those of others) have within the remit of HIV prevention - and public health, generally. This awareness can be effected both at institutional levels through promoting this awareness to staff, but also on a wider scale, such as through relaying these insights through public health and other social media campaigns and marketing strategies.

The insights obtained from this study also indicate that increased levels of funding will have to be allocated towards community-based and other HIV-prevention services to ensure that they are meeting the needs and demands of these communities. Concomitant measures will also require that these services are similarly also made more accessible to these populations.

## References

[ref1] African and Caribbean Council on HIV/AIDS in Ontario *[ACCHO]* (2006). African Black diaspora stream: bringing the hidden epidemic to an international audience.

[ref2] Aronso J. A (1994). Pragmatic view of Thematic Analysis. The Qualitative Report.

[ref3] Black Coalition for AIDS Prevention (Black Cap) (2009). Black Cap report 2009/2010.

[ref4] Centre for Disease Control [CDC] (2007). Centre for Disease Control and Prevention General Considerations Regarding Health Education & Risk Reduction Activities.

[ref5] City of Toronto Backgrounder (2006). Release of the 2006 census on ethnic origin and visible minorities.

[ref6] Edwards C, Titchen A (2003). Research into patients’ perspectives: Relevance and Usefulness of Phenomenological Sociology. Journal of Advanced Nursing.

[ref7] Ezzy D, Rice P (1999). Qualitative Research Methods: A Health Focus.

[ref8] Falconer D. A (2008). Taking Action on HIV and AIDS in Black Communities in Canada: A Resource for Moving Ahead.

[ref9] Fereday J, Muir-Cochrane E (2006). Demonstrating rigor using thematic analysis: a hybrid approach of inductive and deductive coding and theme development. International Journal of Qualitative Methods.

[ref10] HIV Prevention (2009). Global HIV Prevention: The access, funding, and leadership gaps. The Global HIV Prevention Working Group. Fact Sheet.

[ref11] Interagency task team on HIV and young people (2008). Guidance Brief. Community-based HIV interventions for young people. United Nations Population Fund (UNFPA), International Labour Organization (ILO), The Joint United Nations Programme on HIV/AIDS (UNAIDS) Secretariat, United Nations Development Programme (UNDP), The United Nations Economic, Scientific and Cultural Organization (UNESCO) UNESCO, The United Nations High Commission on Refugees (UNHCR), The United Nations Children's Fund (UNICEF), United Nations Office on Drugs and Crime (UNODC), the World Bank, World Food Programme (WFP), World Health Organization (WHO).

[ref12] Koch T (1996). Implementation of a hermeneutic inquiry in nursing: Philosophy, Rigour, and Representation. Journal of Advanced Nursing.

[ref13] Koch T, Harrington A (1998). Reconceptualising rigour: The case for reflexivity. Journal of Advanced Nursing.

[ref14] Leininger M. M, Morse J. M (1994). Evaluation criteria and critique of qualitative research studies. Critical issues in qualitative research methods.

[ref15] Lincoln Y. S, Guba E. G, Denzin N. K, Lincoln Y. S (2000). Paradigmatic controversies, contradictions, and emerging confluences. Handbook of qualitative research.

[ref16] Minichiello V, Madison J, Hays T, Courtney M, St. John W, Minichiello V, Sullivan G, Greenwood K, Axford R (1999). Collecting and evaluating evidence: Qualitative interviews. Handbook for research methods in health sciences.

[ref17] Morse J. M, Field P. A (1995). Qualitative research methods for health professionals.

[ref18] Ng M, Gakidou E, Levin-Rector A, Khera A, Murray C. J. L, Dandona L (2011). Assessment of population-level effect of Avahan, an HIV-prevention initiative in India. Lancet.

[ref19] Schultz A (1967). The Phenomenology of the social World.

[ref20] Seale C (1999). The Quality of Qualitative Research.

[ref21] Seers K, Toye F (2012). What is quality in qualitative health research?. Evidenced Based Nursing.

[ref22] Silverman D (2001). Doing Qualitative Research: A Practical Handbook.

[ref23] Statistics Canada (2012). Toronto, Ontario (Code 3520005) and Ontario (Code 35) (table). Census Profile. 2011 Census. Statistics Canada Catalogue no. 98-316-XWE. Ottawa. http://www12.statcan.ca/census-recensement/2011/dp-pd/prof/index.cfm?Lang=E.

[ref24] Titchen A (2000). Professional craft knowledge in patient-centred nursing and the facilitation of its development.

[ref25] Titchen A, McIntyre D, Titchen A (1993). A phenomenological approach to qualitative data analysis in nursing research. Changing nursing practice through action research.

[ref26] Toronto Public Health (2006). Toronto Takes Action on AIDS.

[ref27] UNAIDS (2005). Intensifying HIV prevention. UNAIDS policy position paper.

[ref28] UNAIDS (2011). World AIDS Day Report. How to get to zero: faster, smarter, better.

[ref29] United Nations General Assembly [UNGASS] (2008). Country Progress Report –CANADA –Government of Canada Report to the Secretary General of the United Nations on the United Nations General Assembly Special Session on HIV/AIDS Declaration of Commitment on HIV/AIDS January 2006 – December 2007. http://data.unaids.org/pub/Report/2008/canada_2008_country_progress_report_en.pdf.

[ref30] Wahoush E (2009). Reaching a hard-to-reach population such as asylum seekers and resettled refugees in Canada. Bulletin World Health Organization.

